# Sequential CD19 and BCMA‐specific CAR T‐cell treatment elicits sustained remission of relapsed and/or refractory myeloma

**DOI:** 10.1002/cam4.3624

**Published:** 2020-12-23

**Authors:** Lingzhi Yan, Su Qu, Jingjing Shang, Xiaolan Shi, Liqing Kang, Nan Xu, Mingqing Zhu, Jin Zhou, Song Jin, Weiqin Yao, Ying Yao, Guanghua Chen, Huirong Chang, Xiaming Zhu, Lei Yu, Depei Wu, Chengcheng Fu

**Affiliations:** ^1^ The First Affiliated Hospital of Soochow University Jiangsu Institute of Hematology National Clinical Research Center for Hematologic Diseases Suzhou China; ^2^ Institute of Blood and Marrow Transplantation Collaborative Innovation Center of Hematology Soochow University Suzhou China; ^3^ Shanghai Unicar‐Therapy Bio‐medicine Technology Co., Ltd. Shanghai China; ^4^ Institute of Biomedical Engineering and Technology Shanghai Engineering Research Center of Molecular Therapeutics and New Drug Development School of Chemistry and Molecular Engineering East China Normal University Shanghai China

**Keywords:** chimeric antigen receptor T (CAR T) cell, dose‐escalation, efficacy, multiple myeloma, relapsed and/or refractory, safety

## Abstract

The low rate of durable response against relapsed and/or refractory multiple myeloma (RRMM) in recent studies indicates that chimeric antigen receptor T‐cell (CART) treatment is yet to be optimized. This study aims to investigate the safety and efficacy of sequential infusion of CD19‐CART and B‐cell maturation antigen (BCMA)‐CARTs for RRMM with a similar 3 + 3 dose escalation combined with a toxicity sentinel design. We enrolled 10 patients, among whom 7 received autologous infusion and 3 received allogeneic infusion. The median follow‐up time was 20 months. The most common grade 3/4 treatment‐emergent toxicities were hematological toxicities. Cytokine‐release syndrome (CRS) adverse reactions were grade 1/2 in 9 out of 10 subjects. No dose‐limited toxicity (DLT) was observed for BCMA‐CAR‐positive T cells ≤5 × 10^7^/kg), while two patients with dose‐levels of 5–6.5 × 10^7^/kg experienced DLTs. The overall response rate was 90% (five partial responses and four stringent complete responses). Three out of four patients with stringent complete responses to autologous CART had progression‐free survival for over 2 years. The three patients with allogeneic CART experienced disease progression within 2 months. These results evidence the sequential infusion's preliminarily tolerability and efficacy in RRMM, and present a simple and safe design applicable for the establishment of multiple CART therapy.

## Graphical abstract

We conducted a pilot study to assess the feasibility of the sequential infusion of CD19 and B‐cell maturation antigen (BCMA)‐specific chimeric antigen receptor T‐cell (CART) for relapsed and/or refractory multiple myeloma treatment with a similar 3 + 3 dose escalation design. Translational Significance: The favorable and persistent responses against RRMM were observed in those patients with a combined infusion of autologous CD19‐CART and BCMA‐CART. The emergence of intolerant adverse event (neurotoxicity and sever CRS) was associated with increased dose level of BCMA‐CARTs infusion.

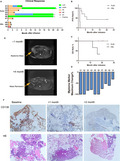

## 1 INTRODUCTION

Currently, patients with refractory and/or relapsed multiple myeloma (RRMM) have limited therapeutic options, and therefore, limited chances to reach the remission plateau. Thus, there is an urgent need for novel RRMM treatment strategies. Recent clinical studies have demonstrated the potential of chimeric antigen receptor‐transduced T cells (CART) that specifically recognize and eradicate tumor cells in a major histocompatibility complex‐independent manner to treat B‐cell malignancy.^1^, ^2^, ^3^, ^4^, ^5^, ^6^, ^7^ B‐cell maturation antigen (BCMA)‐specific CARTs have been effective against RRMM in clinical studies, demonstrating that BCMA is an ideal target of CART therapy for MM.^1^, ^2^, ^4^, ^6^ Furthermore, B‐lymphocyte antigen CD19, which is expressed by B cells prior to terminal differentiation into plasma cells, is associated with the enhancement of myeloma's tumor‐propagating and drug‐resistance properties. Therefore, CD19 is regarded as a potential biomarker of myeloma stem cells and a therapeutic target of MM.^5^, ^8^, ^9^ The sequential expression of CD19 and BCMA in MM tumor cell development suggests that targeting the two biomarkers simultaneously with CART could be a practical strategy to achieve a more efficient response against MM. A treatment utilizing sequential CD19 and BCMA‐specific CART infusion has exhibited promising results in a proof‐of‐concept trial involving RRMM patients.^10^ However, the unavailability of well‐accepted methods and paradigms in dose and regimen selection represents a current challenge in the development of combinatorial CART treatment.

This pilot clinical trial with a toxicity sentinel design and a dose escalation approach was, therefore, conducted to assess the safety and feasibility of the sequential infusion of CD19 and BCMA‐specific CARTs for RRMM treatment.

## 2 MATERIALS AND METHODS

### 2.1 Trial design

This study was approved by the Ethics Committee of the First Affiliated Hospital of Soochow University, P.R. China in December 2016 and registered with ClinicalTrials.gov (NCT 03196414). Because of safety concerns for the first application of BCMA‐CART in the hospital, the number of patients enrolled in this study was limited to 10. Ten patients with refractory/relapsed BCMA‐positive myeloma were enrolled in the study after providing informed consent. Subjects were considered eligible if they met the following criteria: ≥18 years of age, documented evidence of drug resistance and/or progression of disease (PD) as defined by the International Myeloma Working Group (IMWG) criteria^11^ after completing at least one prior regimen, ≥4 weeks washout period prior to CART infusion since their latest cytotoxic chemotherapy treatment, an ECOG performance status score of 0–2, and an expected survival time of ≥3 months. The exclusion criteria included: clinically significant cardiac disease, serum creatinine ≥2 mg/dl, active infection, seropositive HIV, and HBS‐Ag, HCV‐Ab, and/or HBV‐DNA or HCV‐RNA above the negative level. Patients with plasma cell leukemia were also excluded.

To investigate whether unexpected adverse events would occur with the infusion of CART‐BCMA, which had not been used before in the hospital, we included a sentinel subject (Patient 01) who first received a single dose of CART‐BCMA and received a combinatorial treatment with CART‐CD19 and CART‐BCMA around 5 weeks later. Then, the trial entered the dose escalation phase. Qualified patients were sequentially assigned to three cohorts and administered a fixed dose of 1.0 × 10^7^/kg CD19‐CAR‐positive T cells combined with increased dose levels of approximately 3.0, 5.0, and 6.5 × 10^7^/kg BCMA‐CAR‐positive T cells, respectively. Dose escalation conducted in the study was similar to a classic 3 + 3 design in order to explore the optimal dose level of BCMA‐CARTs in the context of dual CART treatment. Decision‐making criteria for dose‐escalation were as follows:

- If none of the three patients in the preceding cohort experiences a dose‐limiting toxicity (DLT), another three patients will be assigned to the next cohort.
- If one of the three patients in the cohort experiences a DLT, another three patients will be assigned to the same cohort.
- The dose escalation stops if two or more patients within a cohort experience DLTs. The rest of the patients will be assigned to the preceding cohort.
- The trial is terminated once a total of 10 patients have been dosed with CAR T cells.
- DLT’s are defined as neurotoxicity and severe CRS requiring any clinical intervention.

The primary end point was the incidence of treatment emergent adverse events, including adverse events (AEs), serious adverse events (SAEs), deaths, or laboratory abnormalities. AEs and SAEs occurring in the first 2 weeks and 2 weeks after the infusion are defined as acute and late, respectively, and reported separately. Secondary end points were efficacy against MM, including response rate and duration. Exploratory end points included independent evaluation of response, overall survival (OS) and progression‐free survival rate, measurement of serological biomarkers, and kinetics of CARTs in vivo.

### 2.2 Treatment schedule and administration

**FIGURE 1:**
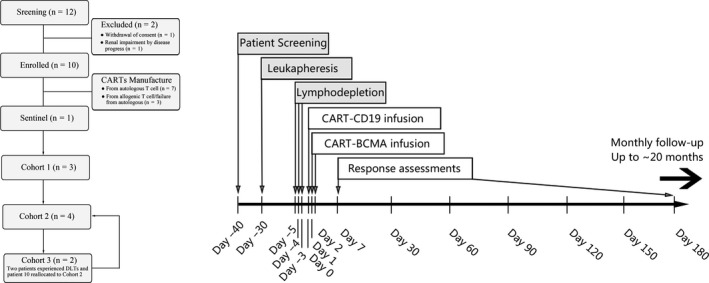
CONSORT diagram and scheme of the clinical trial design. Following patient enrollment, autologous or allogeneic T cells were obtained via leukapheresis and transfected to generate CART‐CD19 or CARTBCMA. After administering short‐term chemotherapy for lymphodepletion (3 doses of cyclophosphamide and fludarabine), patients received one‐dose infusion of CART‐CD19 on day 0 and then a split‐dose infusion of CART‐BCMA over 2 days (40% on day 1 and 60% on day 2). Patients were admitted for 2 weeks for management of potential adverse events, followed by long‐term follow‐up for response assessment. All patients reported here were infused with CAR T cells between July 2017 and November 2017. The presented data cut‐off date was July 31, 2019.

All of the patients received daily doses of 300 mg/m^2^ cyclophosphamide and 30 mg/m^2^ fludarabine for leukocyte depletion on days 5, 4, and 3 prior to CART infusion. CD19‐CART was infused at a fixed dose of 1.0 × 10^7^/kg body weight on day 0. Patients including the sentinel also received a split‐dose of BCMA‐CART infusion, with 40% given on day 1 and 60% on day 2. Three dose‐levels of BCMA‐CART (Level 1 approximately 3.0 × 10^7^/kg, Level 2 approximately 5 × 10^7^/kg and Level 3 approximately 6.5 × 10^7^/kg), were administered to patients. The treatment schedule protocol is schematically presented in Figure 1 and CART infusion is detailed in Table 1.

**TABLE 1:** Patients’ characteristics, cell infusion dose, and in vivo CAR T‐cell persistence.

Patient^a^	Age/gender	Monoclonal protein	Prior lines of therapy	Prior ASCT	High‐risk chromosome^b^	Relapse classification^c^	BCMA(%) on myeloma cells	T cell source	Infusion dose (per kg)^d^ CART‐CD19 CART‐BCMA		Dose level^e^	CART expansion duration (days)^f^
Sentinel 01	64/M	IgG‐κ	4	Y	t(4;14)	Aggressive	91.5	Autologous	N/A	3.2 × 10^7^	‐	‐
									1 × 10^7^	5 × 10^7^		
03	57/M	IgG‐κ	3	Y	Normal	Aggressive	81.6	Autologous	1 × 10^7^	3.0 × 10^7^	1	63
04	47/F	IgG‐λ	4	Y	Normal	Extramedullary	69.7	Autologous	1 × 10^7^	2.5 × 10^7^	1	>106
05	69/M	IgG‐λ	2	N	1q21 amp	Aggressive	96.9	Autologous	1 × 10^7^	2.5 × 10^7^	1	7
02	43/M	IgG‐κ	4	Y	Normal	Extramedullary	54.2	Autologous	1 × 10^7^	4.5 × 10^7^	2	8
07	57/M	IgD‐λ	3	N	NA	Aggressive	92.5	Autologous	1 × 10^7^	5.0 × 10^7^	2	>12
09	58/M	IgA‐κ	5	Y	1q21 amp	Aggressive	94.6	Allogenic	1 × 10^7^	4.75 × 10^7^	2	10
06	60/M	IgG‐κ	3	N	Normal	Biochemical	80.8	Autologous	1 × 10^7^	6.2 × 10^7^	3	>98
08	60/F	IgG‐κ	7	N	t(4;14)	Aggressive	55.7	Allogenic	1 × 10^7^	6.8 × 10^7^	3	>36
10	48/F	IgG‐κ	3	Y	1q21 amp	Aggressive	88	Allogenic	1 × 10^7^	4.6 × 10^7^	2	>8

Abbreviations: ASCT, autologous stem cell transplant; BCMA, B‐cell maturation antigen; CART, chimeric antigen receptor T cells; N/A, unavailable.

^a^ Patient is listed sequentially in the order that they received CART infusion and Patient number was given in the order that they were enrolled in the trial (signed Informed Consent Form)

^b^ Detection of cytogenetics in clonal plasma cells from bone marrow aspirate performed prior to CAR T‐cell infusion. The patient 07 had no cytogenetic or FISH results.

^c^ Definition of relapse classification was according to the IMWG criteria. “Biochemical relapse” was considered if it presented with asymptomatic increase in the M‐component only. “Aggressive relapse” was considered if it presented with at least one of the following features: doubling of M protein rate over 2 months, renal insufficiency, hypercalcemia, extramedullary disease, elevated LDH, presence of plasma cells in peripheral blood, or skeletal‐related complications. “Extramedullary relapse” was considered if it presented with a large extramedullary mass as the main recurrent lesion.

^d^ Patient 01 used as the sentinel subject received an infusion of CART‐BCMA followed by the combinatorial treatment with CART‐CD19 and CART‐BCMA, regarding the risk from the sequential infusion of the two types of CAR T‐cell.

^e^ Except the sentinel subject, nine patients were assigned to three subgroups with different dose levels of CART‐BCMA and a fixed dose of CART‐CD19.

^f^ CART expansion duration spans the time of the first day of CAR T‐cells infusion until the last day with the CAR transgene copies over baseline.

### 2.3 Safety and response assessment

Peripheral blood (PB) samples were obtained to perform safety and response assessments at predetermined time points. The IMWG Response Criteria for Multiple Myeloma was used to assess the response to treatment.^11^ Minimal residual disease (MRD) was assessed by a 10‐color multiparameter flow cytometry assay with a minimum cutoff of 2 × 10^−6^ nucleated cells (Beckman Coulter, Beckman Coulter NAVIOS). Cytokine release syndrome (CRS) and neurotoxic adverse effects were graded and managed according to the recommendations of Lee et al.^12^ Other toxicities were assessed according to the National Cancer Institute's Common Terminology Criteria for Adverse Events, version 4.03.^13^ Plasma levels of IL‐2, IL‐4, IL‐6, IL‐10, (Interleukin) TNF‐α, (Tumor necrosis factorα) IFN‐γ, (Interferon‐γ) and IL‐17A were determined using the BD Cytometric Bead Array Human Th1/Th2/Th17 kit (BD). The blood concentration of serum free BCMA was evaluated using the Cytometric Bead Array BCMA kit (QuantoBio).

### 2.4 CART production and detection

Chimeric antigen receptor T‐cell production, blood sample processing, and laboratory analyses were performed by the Unicar‐Therapy Bio‐medicine Technology Company. In brief, T cells were purified from PB mononuclear cells using microbeads (Miltenyi Biotec) and were stimulated with an anti‐CD3 monoclonal antibody. Stimulated T cells were transduced with a recombinant lentiviral vector encoding the BCMA or CD19‐specific CAR, the constructions of which are presented in Figure S1. The detailed manufacturing protocol and analyses of these samples are provided in the Supporting Information including Figure S2. For the 3 out of 10 subjects whose autologous T cells did not undergo successful CART engineering, CART products were generated from T cells from a related allogeneic donor who was a 5/10 loci haploidentical match as determined by detection of HLA‐A, B, Cw, DRB1, and DQB1 loci using high‐resolution genotyping. To determine the in vivo expansion and persistence of CARTs in the PB, qPCR (quantitative Polymerase Chain Reaction) was performed on DNA isolated from patients’ blood samples to detect the frequency of total CARTs.

## 3 RESULTS

### 3.1 Patient characterization

Ten RRMM patients with advanced stage disease were enrolled in the study. All 10 patients showed BCMA expression in either clonal bone marrow (BM) or PB plasma cells with a positive rate of over 50%, and none of the patients expressed CD19, as determined by flow cytometry. The patients had received a median of four prior lines of therapy, and 60% had received an autologous stem cell transplant prior to CART therapy (Table 1). Furthermore, 90% of the patients were refractory to proteasome inhibitors, immunomodulatory drugs, or both (Table S1). The median absolute lymphocyte count in the PB at the time of leukapheresis was 1120 cells/μl (range: 250–1690) for seven patients (Patients 01–07) with autologous CART infusions. Three patients (Patients 08, 09, and 10) received infusions of allogeneic CARTs derived from related haploidentical donors whose lymphocyte counts were within the normal range. Patient characterization is detailed in Table 1. Patient number was given in the order that they were enrolled in the trial (signed Informed Consent Form). Patient is listed sequentially in the order that they received CART infusion. As it took different time for CAR T‐cell preparation between patients. CAR T‐cells administration was not conducted in the order of number.

### 3.2 CART toxicity

Adverse events after CART infusion were categorized as follows: early acute stage reactions (≤2 weeks) and late stage reactions (>2 weeks). Toxic reactions involved hematological and non‐hematological aspects, including CRS and CAR‑T‑cell‑related encephalopathy syndrome (CRES) toxicities. Comparing the safety profiles of the sentinel subject after the first and second infusions, there were no unexpected AEs with sequential infusion of CART‐CD19 and CART‐BCMA.

Although CRS was observed in 100% of patients, grade 3 (severe) toxicity was only noted in Patient 06. There were no cases of CRS of grade 4 or higher (Table 2). Furthermore, patient 08 experienced grade 2 CRS and grade 1 CRES, which was limited to confusion (Table 2). Patients 01 and 09 (20%) suffered from grade 3 tumor lysis syndrome, which resolved following intravenous fluid treatment. A higher maximal value of CAR T‐cell expansion was also noted in patients with severe CRS. No patients received corticosteroids, and none required mechanical ventilation. However, patients with persistent fever and a high level of IL‐6 (Patients 01 and 06) were treated with the systemic anti‐IL‐6 receptor monoclonal antibody tocilizumab, which resulted in a rapid resolution of symptoms. Additionally, Patient 09 was also the only one who suffered a systemic infection of *Staphylococcal septicaemia* during the treatment process. Given that two patients at dose level 3 both experienced prespecified DLTs, the dose of BCMA‐CART for Patient 10 was reverted to the preceding dose level according to the 3 + 3 rule.

**TABLE 2:** CRS, CRES, and AEs in different dose groups of CARTs‐BCMA.

CARTs‐BCMA	~3.0 × 10^7^/kg (n = 3)			~5.0 × 10^7^/kg (n = 4)			~6.5 × 10^7^/kg (n = 2)		
AEs	Grade1‐2	Grade3	Grade4	Grade1‐2	Grade3	Grade4	Grade1‐2	Grade3	Grade4
CRS	3 (100%)	0	0	4 (100%)	0	0	1 (50%)	1 (50%)	0
CRES	0	0	0	0	0	0	1 (50%)	0	0
Hematological AE									
Neutropenia	3 (100%)	0	0	1 (25%)	1 (25%)	2 (50%)	0	1 (50%)	1 (50%)
Leukopenia	0	0	3 (100%)	0	0	4 (100%)	0	0	2 (100%)
Anemia	3 (100%)	0	0	2 (50%)	2 (50%)	0	1 (50%)	1 (50%)	0
Thrombocytopenia	2 (67%)	0	0	1 (25%)	3 (75%)	0	0	0	2 (100%)
Non‐hematological AE									
Fever	3 (100%)	0	0	3 (75%)	1 (25%)	0	0	1 (50%)	1 (50%)
Fatigue	3 (100%)	0	0	4 (100%)	0	0	0	1 (50%)	1 (50%)
Nausea	1 (33%)	0	0	2 (50%)	0	0	2 (100%)	0	0
Vomiting	0	0	0	2 (50%)	0	0	2 (100%)	0	0
Diarrhea	1 (33%)	0	0	2 (50%)	0	0	2 (100%)	0	0
Myalgias	1 (33%)	0	0	3 (75%)	0	0	2 (100%)	0	0
Redness flush	2 (67%)	0	0	1 (25%)	0	0	2 (100%)	0	0
Bilirubin elevated	0	0	0				2 (100%)	0	0
Transaminase increased	1 (33%)	0	0	3 (75%)	0	0	2 (100%)	0	0
Creatinine increase	1 (33%)	0	0	1 (25%)	0	0	1 (50%)	0	0
Prolonged APTT	0	0	0	2 (50%)	0	0	0	2 (100%)	0
TLS	0	0	0	0	1 (25%)	0	0	0	0
NT‐proBNP elevated	2 (67%)	0	0	1 (25%)	2 (50%)	0	0	2 (100%)	0
Sepsis	0	0	0	1 (25%)	0	0	0	0	0
Hypotension	0	0	0	1 (25%)	0	0	2 (100%)	0	0
Hypoxia	0	0	0	2 (50%)	0	0	2 (100%)	0	0

Note

All patients in this trial also received a fixed dose of 1 × 10^7^/kg CART‐CD19 cells before different dose of CART‐BCMA infusion. Data are listed for events from Patient 02–10. CRS was graded according to recommendations by Lee et al and CRES was graded according to recommendations of the CAR‐T cell‐therapy‐associated toxicity (CARTOX) Working Group. Other toxicities were assessed according to the National Cancer Institute Common Terminology Criteria for Adverse Events, version 4.03.

Abbreviations: AE, adverse event; APTT, activated partial thromboplastin time; CRES, CAR‑T‑cell‑related encephalopathy syndrome; CRS, cytokine release syndrome; NT‐proBNP, N terminal pro B type natriuretic peptide;TLS, tumor lysis syndrome.

Severe cytopenia developed in 80% of the patients after infusion and chemotherapy, and anticipatory lymphocyte depletion was observed in all of the patients, which all resolved within 1 week. Patient 08 required a platelet transfusion for thrombocytopenia. None of the patients were treated with colony‐stimulating factor, erythropoietin, or thrombopoietin agents. APTT elongation occurred in 50% of the patients and was resolved with repeated plasma infusion. Only 20% (2/10) patients developed mild bleeding of the skin and bulbar conjunctiva. The hematological and non‐hematological toxicities in three different dose groups are shown in Table 2.

No fatal or SAEs emerged after 2 weeks following the infusion. Notably, Patient 02 had grade 1 chronic diarrhea and Patient 04 had a grade 2 soft tissue infection following local trauma. Furthermore, 50% of the patients experienced grade ≤2 bilirubin and/or transaminase elevation, and 40% of the patients experienced a minor transient upper respiratory infection. Gamma globulin infusions were administered to 40% of the patients to boost their immunity against potential infections. All of the acute and late AEs are shown in Table S2.

### 3.3 Clinical response

**FIGURE 2:**
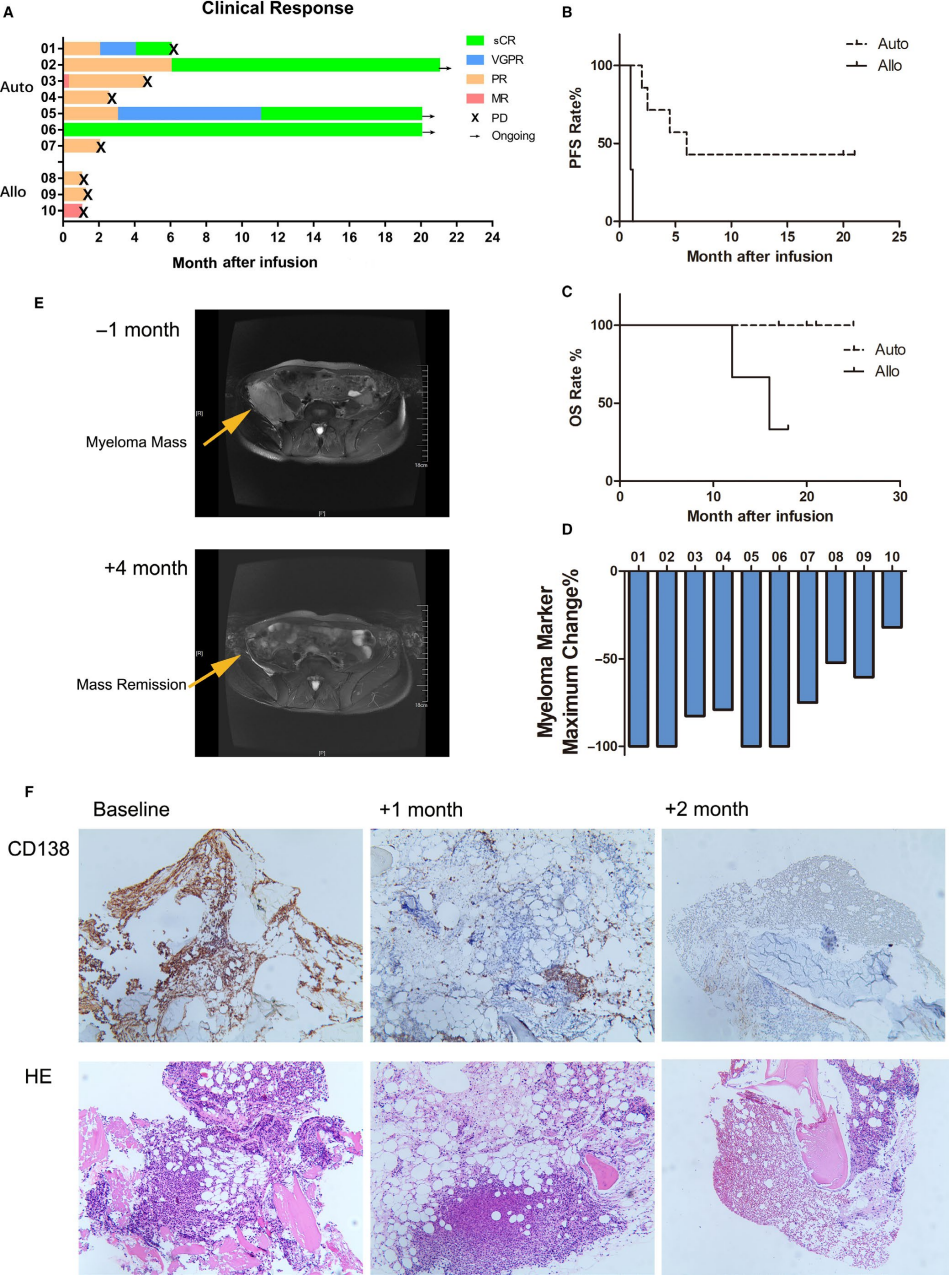
Sequential CD19‐ and BCMA‐CART treatment induced an anti‐tumor response in MM patients. A, The swimmer's plot shows the treatment response and response duration for patients administered the autologous or allogeneic infusions. The depth of anti‐myeloma response is indicated by different colors. × indicates the time point when progression of disease occurred. → indicates clinical response persisted at the last follow‐up. B, Kaplan–Meier curve of progression‐free survival (PFS) in patients with autologous or allogeneic CART treatment. Three patients who received autologous CART treatment remained in ongoing response at the last follow‐up. C, Kaplan–Meier curve of overall survival (OS) in patients receiving autologous or allogeneic CART treatment. One patient who received allogeneic CART and all seven patients who received autologous treatment were still alive at the last follow‐up. D, The waterfall plot represents the maximum percentage change in the myeloma marker serum immunoglobulin M protein post infusion. E, Diffusion weighted magnetic resonance imaging (DW‐MRI) of patient 02's right iliac fossa a month prior to and 4 months after CART infusion. F, Immunohistochemistry staining of Patient 01's BM biopsy with CD138 indicated that BM myeloma dramatically decreased 1 month after CART infusion and remained at low levels 2 month after the infusion.

The overall response rate (ORR) (defined as at least a partial response, PR) was 90% (9/10), with 100% of the patients experiencing a clinical benefit (ORR + minimal response, MR). Furthermore, 57% of the patients (4/7) who received autologous CART infusion demonstrated stringent complete response (sCR). Three out of these four patients with sCR showed an absence of clonal plasma cells in BM by multiparametric flow cytometry (with 10 colors). This response was maintained until the follow‐up cut‐off date (July 31, 2019). In contrast, the three patients who received allogeneic CART infusion demonstrated PR or MR at best, and two patients died of PD at approximately 1 year after the infusion. The swimmer's plot shows the depth of response against MM and the duration of progression‐free survival (PFS) for each patient (Figure 2A). Responses better than PR were associated with persistent PFS. Notably, the PFS and OS rates of the patients who received autologous CARTs were higher than the rates of those who received allogeneic CARTs (Figure 2B,C). The difference in the effect between autologous and allogeneic CART infusion was also indicated by the maximum change in serum myeloma protein concentrations (Figure 2D). Of note, Patient 02 developed a myeloma extramedullary mass located in the right iliac fossa 4 months after infusion (Figure 2E). The effect of CART infusion on plasma cell eradication in the BM was demonstrated by a combination of flow cytometry and immunohistochemistry staining of biopsies for CD138. Notably, the BM biopsy of Patient 01 demonstrated a gradual depletion of plasma cells within 2 months postinfusion (Figure 2F).

### 3.4 High expansion of CAR T‐cells in vivo was associated with clinical activity

**FIGURE 3:**
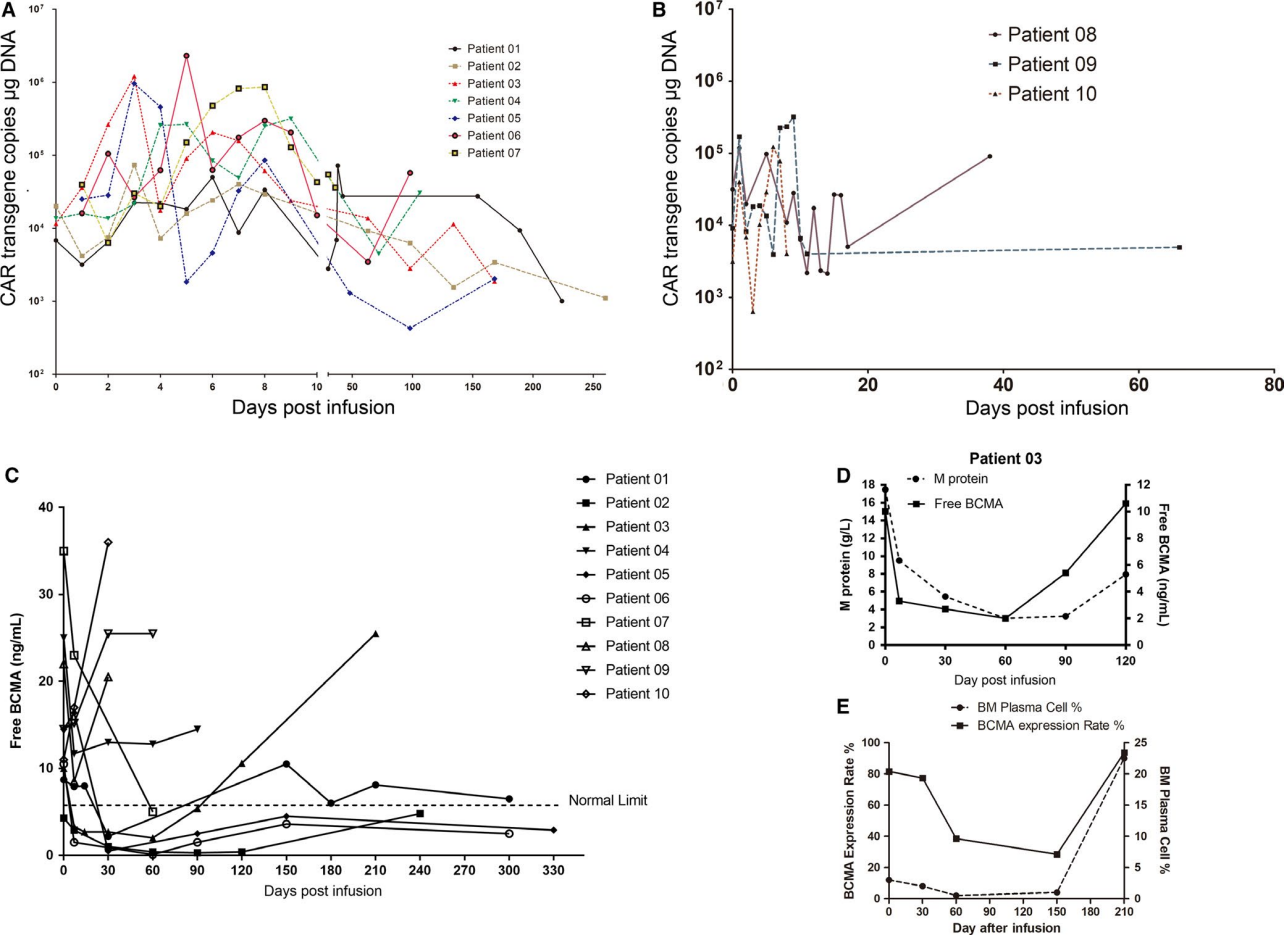
Response against myeloma by sequential CD19‐ and BCMA‐CART treatment was associated with a high dose of autologous CART infusion and decreased BCMA expression. A and B, The copies of total CAR transgene in genomic DNA extracted from peripheral blood mononuclear cells were determined by quantitative polymerase chain reaction and used to describe in vivo CART cell expansion in patients who received autologous (A) CARTs or allogeneic (B) CARTs. C, Dynamics of free BCMA in patients' serum was determined by enzyme‐linked immunosorbent assay. Serum BCMA concentration remained under normal limit in patients with durable remissions. D, The curve of free BCMA concentration in peripheral blood (PB) was similar to the change in M protein post CART cell infusion for patient 03. E, BCMA antigen expression on bone marrow (BM) plasma cells from patient 03 evaluated with multiple‐color flow cytometry. BCMA expression initially decreases with response following CART therapy, then increases at progression

The patients who received autologous and high‐dose infusions exhibited a higher magnitude of in vivo expansion and complete antimyeloma response (Patients 01 and 06; Figure 3A). In contrast, the administration of allogeneic CART was associated with low peak CAR transgene copies and poor persistence (approximately 8 days) (Figure 3B). In most cases, the PB levels of CAR transgene copies returned to baseline by 1 month postinfusion, except in Patient 06. The rapid loss of persistent CARTs in the PB may have contributed to Patient 06’s MM relapse. Intensive total CAR T‐cell expansion, defined as a maximum value of 100‐fold higher than that on day 1, was detected in blood samples from the two patients with CR (Patients 01 and 06), suggesting that CAR T‐cell early expansion postinfusion may be predictive of clinical outcome.

### 3.5 BCMA‐CART dose escalation was associated with more intense toxicity

The toxic signals captured from the sentinel subject showed that BCMA‐CART did not result in any unexpected AEs in the presence or absence of CD19‐CART. However, we observed exacerbated CART‐relevant AEs in subjects who received a high dose of BCMA‐CART, especially in those who received level 3 of dose‐escalation. Severe CRS, fever, and increase in NT‐proBNP (grade 3) were observed in Patient 06 with 6.2 × 10^7^/kg BCMA‐CART, while severe fever (grade 4), increase in NT‐proBNP (grade 3), CRS (grade 2), and CRES (grade 1) emerged in Patient 08 with 6.8 × 10^7^/kg BCMA‐CART (Table S2).

### 3.6 Serum BCMA expression kinetics were associated with MM status postinfusion

A previous study indicated that serum BCMA level was an effective indicator of disease status and therapeutic response in patients with MM.^14^ Accordingly, in patients who received autologous CARTs, the decline in serum‐free BCMA simultaneously occurred with therapeutic response against myeloma (Figure 3C). Persistent serum BCMA at a low level was observed in those who had durable clinical responses (Patients 02, 05, and 06). However, the infusion of allogeneic CART failed to suppress serum BCMA. Of note, Patient 03’s serum BCMA level decreased following CART infusion and rebounded to baseline level or higher by 3 months, concomitant with the increasing serum M protein level (Figure 3D). Additionally, surface BCMA antigen expression on BM plasma cells, as determined by flow cytometry, initially declined following CART infusions, then, ascended to a level even higher than baseline at relapse (Figure 3E).

### 3.7 CD19‐CART/BCMA‐CART infusion induces a rapid immune response to MM

**FIGURE 4:**
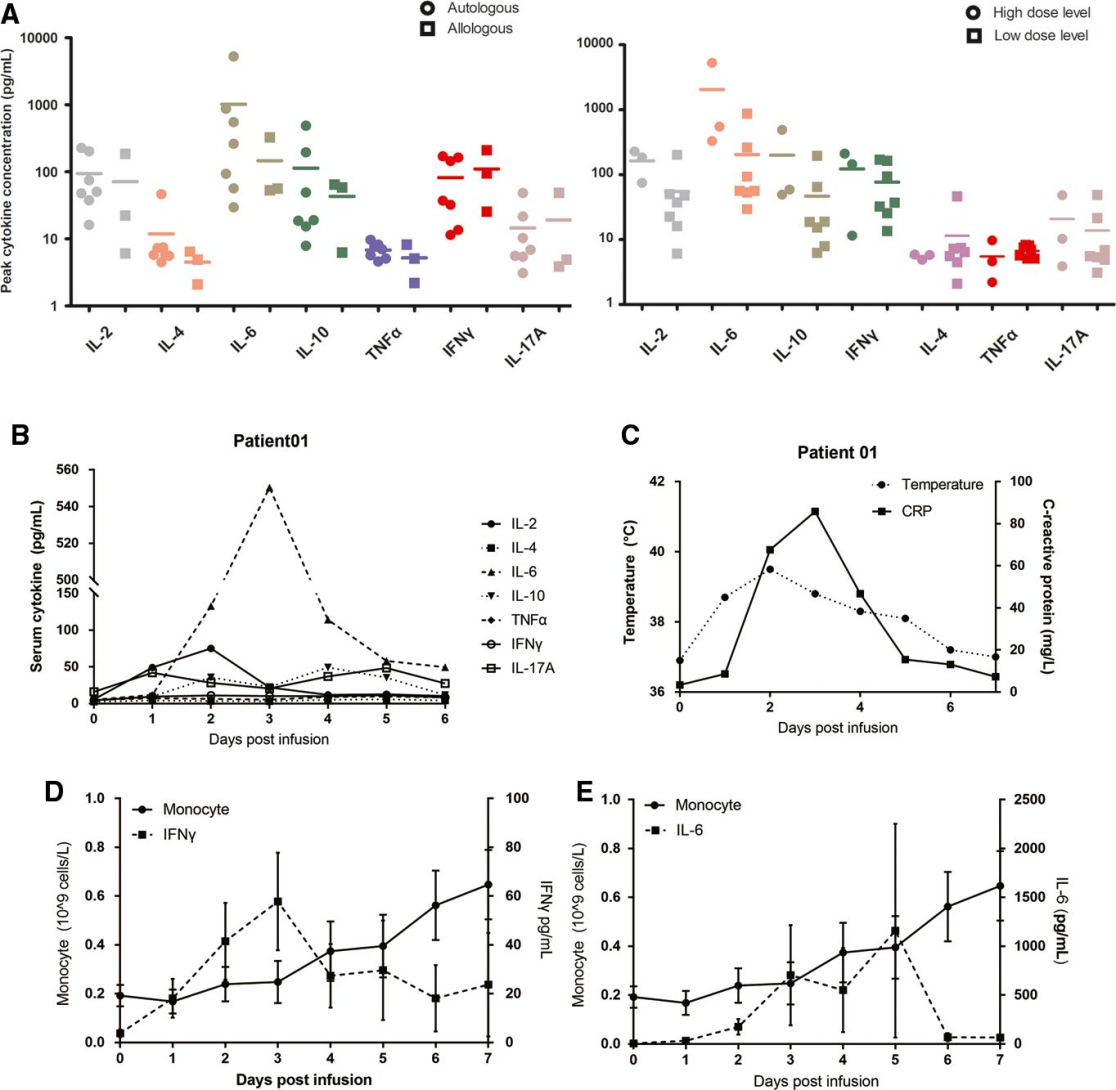
Autologus and Allologous CD19‐CART/BCMA‐CART cells infusion induced a rapid immune response in MM patients. A, Serum concentrations of a panel of seven cytokines were determined within the first week after CART‐BCMA infusion using a multiplexed particle‐based flow cytometric assay. The peak serum concentrations of each of the seven cytokines in the autologous and allogeneic groups are presented (left panel). The maximum concentration of four cytokines (IL‐2, IL‐6, IL‐10, and IFNγ) increased more in patients receiving a high dose of CART‐BCMA vs. those with low dose (right panel). B and C, The time course changes in cytokine levels in the blood, temperature and serum C‐reaction protein level of Patient 01. D and E, The kinetics of mean (+*SEM*) monocyte count is presented compared with mean (+*SEM*) IFNγ(D) or IL‐6 (E) level.

The kinetics of cytokine production in PB were measured during the first week postinfusion. The three patients who received high‐dose BCMA‐CART (levels 3 and sentinel) exhibited higher serum cytokine peak values (Figure 4A), suggesting a correlation between CAR T‐cell input and cytokine production. Throughout this period, the concentration of the seven cytokines initially increased; however, all cytokine concentrations returned to the baseline within 1 week (Figure 4B). Patients with CRS experienced fluctuations in temperature and serum CRP concentration with a pattern similar to that of IL‐6 concentrations (Figure 4C). Furthermore, when monocyte counts were not yet recovered from conditioning chemotherapy, we observed that an increase in T cell activation indicator IFNγ and CRS indicator IL‐6 occurred (Figure 4D,E), which may contribute to CRS of mild intensity and a short duration in these patients (range: 1–9 days) in the trial.

## 4 DISCUSSION

New immunotherapeutic options are critical for patients with drug‐resistant RRMM. BCMA‐specific CART treatment is a promising option for these patients, demonstrating superior response rates, but recent studies show that most responsive patients eventually relapse.^1^, ^2^, ^3^, ^4^, ^6^, ^15^ Bruno et al. demonstrated that BCMA expression varied greatly among the myeloma cells of different patients, and that nonresponsive patients had tumor cells with lower levels of BCMA expression.^3^ The loss or downregulation of BCMA from myeloma cells of some patients after infusion of BCMA‐CARTs, which corresponded with the onset of myeloma progression, has also been demonstrated.^3^, ^4^ These data suggest that BCMA escape is likely in MM patients and that CART therapy targeting a single antigen may be inadequate for these patients. CD19 plays major roles in human B‐cell lineage differentiation, and its expression generally decreases in terminal plasma cells or myeloma cells.^16^, ^17^, ^18^ Several studies have shown that a small population of less‐terminally differentiated CD19^+^ plasma cells may make up a drug‐resistant, clonogenic disease reservoir that is maintained by components of the BM microenvironment.^19^, ^20^, ^21^ These findings suggest that CD19 is also a promising target when combined with BCMA‐CART treatment.

This study was designed to observe the safety and efficacy of a combined infusion of CD19 and BCMA‐specific CARTs for RRMM. First, we demonstrated that the combined CART infusion was well tolerated in patients with RRMM. Due to the conditioning chemotherapy, hematologic toxicities were common in the study, and they were similar to those observed in other CART clinical treatment studies.^1^, ^2^, ^3^, ^4^, ^6^ Of note, CRS was generally mild in this study, despite the fact that this study used higher doses of CARTs than were used in previous studies. Only one patient experienced grade 3 CRS, but the patient fully recovered within 1 week after receiving one low dose of tocilizumab. Due to the occurrence of DLTs in patients 06 and 08, BCMA‐CART dose escalation was stopped at level 3, and the last subject (patient 10) was reassigned to Cohort 2. This indicates that high‐dose BCMA‐CART treatment is correlated with the critical safety signals of CAR therapy (CRS and CRES). Indeed, the safety issue in this study is mild. Intensive care combined with clinical inventions post‐CART infusion may allow patients to tolerate higher CART dose. However, the trial hardly evidences that BCMA‐CART dose escalation improves the clinical response in RRMM patients. Further studies with larger sample sizes are required to clearly determine whether a dose‐response relationship exists, as well as to establish an optimal strategy balancing safety, efficiency, and clinical expense.

In three of the seven patients treated with auto‐CARTs, sustained negative MRD was noted for more than 20 months. This was consistent with the findings of a recent study on the use of anti‐CD19 and anti‐BCMA CARs in vitro for primary tumor cells from myeloma patients, which showed a consistent, significant reduction in myeloma‐propagating capability.^22^ The favorable responses reported herein support the synergy of a combined infusion of CD19‐CART and BCMA‐CART against RRMM.

High‐quality T cells are the basis for preparing CARTs. In this study, three patients were given allogeneic CARTs due to T‐cell aging and nonproliferation in vitro. Graft‐vs.‐host (GVH) reactions were the priority concern for treatments with CARTs derived from allogeneic T cells. There were no GVH reactions in this study, but a short duration of allogeneic CART persistence and poor clinical response was observed. There are several potential mechanisms underlying the poor efficacy of these allogeneic CART infusions. It has been demonstrated preclinically that co‐activation of CAR and endogenous T cell receptor (TCR) leads to rapid CART exhaustion, and thus, may compromise CART persistence and expansion.^23^ The clinical application of allogeneic CARTs derived from healthy donors may require novel approaches (e.g., gene editing to remove endogenous TCR molecules) to increase their persistency in treatment by preventing cell apoptosis upon overactivation.^24^ In addition, the lymphodepletion regimen used for preconditioning may have been insufficient to prevent host‐mediated immune rejection of the haploidentical donor CARTs, leading to limited cell expansion and anti‐MM efficacy.

Regulatory B cells (Bregs) identified by the CD19+CD24+CD38+ phenotype are an important immunosuppressive components for myeloma, as they prevent immune effector cells from homing and interacting with targeted tumor cells.^25^ Bregs have been demonstrated to inhibit the cytotoxicity of T cells by producing lL‐10 and also to maintain the immunosuppressive microenvironment.^25^, ^26^, ^27^ We hypothesized that combined infusion of CD19‐CARTs could also help eradicate CD19+ B cells, including Bregs, from BM cells, and thereby contribute to reshaping the BM microenvironment. This would indicate their favorability to CART synergism in MM therapy. To address this question, we analyzed the frequency of Bregs within CD19+ B cell population in Patient 05 by flow cytometry due to the lack of samples from other patients. Patient 05’s Bregs accumulated in BM (50.15%), instead of PB (2.4%), prior to the combined CART infusion. However, 1 month post‐infusion, Bregs, along with the total CD19+ B cell population, were eliminated from BM, which is consistent with the patient's disease remission. Furthermore, 6 months post‐infusion, Bregs were still suppressed at a lower proportion (2.5%) even though CD19+B cells in the BM had recovered from clearance by CD19‐CARTs infusion (Figure S3). These results suggest that the elimination of Bregs from the BM is the mechanism underlying the dual CART treatment, and that the detection of BM Bregs may be used to identify eligible subjects for this therapy from the overall RRMM population. However, this relationship between the eradication of Bregs in BM and a lasting clinical response still need to be verified by improved animal models and stronger clinical evidence from large‐scale studies.

It is unclear if the early phase designs of CART drug development that have been proposed for small molecular or biological agents can be readily applied to CARTs whose viability distinguishes them from conventional agents. However, few studies focus on development methods or models specific for CART drugs. Due to the unavailability of animal models applicable for dual CARTs therapy, the starting dose and dose increment designs are drawn from empirical evidence, rather than extrapolation from animal toxicological data. Another limitation of the study is that we did not investigate the immunogenicity of CD19‐CARTs or BCMA‐CARTs, which has a potential impact on efficacy and safety, especially in repeated dosing treatment.

In summary, this study describes the administration of a novel treatment strategy against RRMM using the combination of BCMA‐specific CARTs and CD19‐specific CARTs. RRMM patients in China are minimally treated by global standards due to lack of exposure to the proteasome inhibitor carfilzomib, the immunomodulatory molecules lenalidomide and pomalidomide, and the monoclonal antibody daratumumab. However, this does not detract from our study's results on the feasibility and safety of administering two different CART types sequentially. The sequential infusion was well tolerated and was associated with impressive efficacy against myeloma in RRMM patients. Future prospective and randomized controlled studies are needed to investigate the benefits and risks of co‐infusion compared to those of single‐target CAR T‐cell infusion for MM treatment.

## CONFLICT OF INTEREST

All authors declare that they have no conflict of interest. This manuscript involving the research protocol had been approved by the Ethics Committee of the First Affiliated Hospital of Soochow University and the Helsinki Declaration of 1975, as revised in 2000 (5). Informed consent was obtained from all the enrolled patients in the study.

## Supporting information

Supplementary Material

## Data Availability

The data that support the results of this study are not publicly available but may be made available by the corresponding author upon reasonable request.

## References

[1] Raje N , Berdeja J , Lin YI , et al. Anti‐BCMA CAR T‐Cell therapy bb2121 in relapsed or refractory multiple myeloma. N Engl J Med. 2019;380(18):1726‐1737. 31042825 10.1056/NEJMoa1817226 PMC8202968

[2] Cohen AD , Garfall AL , Stadtmauer EA , et al. B cell maturation antigen‐specific CAR T cells are clinically active in multiple myeloma. J Clin Invest. 2019;129(6):.2210–2221. 30896447 10.1172/JCI126397 PMC6546468

[3] Brudno JN , Maric I , Hartman SD , et al. T cells genetically modified to express an anti‐B‐cell maturation antigen chimeric antigen receptor cause remissions of poor‐prognosis relapsed multiple myeloma. J Clin Oncol. 2018;36(22):2267‐2280. 29812997 10.1200/JCO.2018.77.8084 PMC6067798

[4] Ali SA , Shi V , Maric I , et al. T cells expressing an anti‐B‐cell maturation antigen chimeric antigen receptor cause remissions of multiple myeloma. Blood. 2016;128(13):1688‐1700. 27412889 10.1182/blood-2016-04-711903 PMC5043125

[5] Garfall AL , Maus MV , Hwang W‐T , et al. Chimeric antigen receptor T cells against CD19 for multiple myeloma. N Engl J Med. 2015;373(11):1040‐1047. 26352815 10.1056/NEJMoa1504542 PMC4646711

[6] Xu J , Chen L‐J , Yang S‐S , et al. Exploratory trial of a biepitopic CAR T‐targeting B cell maturation antigen in relapsed/refractory multiple myeloma. Proc Natl Acad Sci USA. 2019;116(19):9543‐9551. 30988175 10.1073/pnas.1819745116 PMC6510991

[7] Ramos CA , Savoldo B , Torrano V , et al. Clinical responses with T lymphocytes targeting malignancy‐associated kappa light chains. J Clin Invest. 2016;126(7):2588‐2596. 27270177 10.1172/JCI86000 PMC4922690

[8] Boucher K , Parquet N , Widen R , et al. Stemness of B‐cell progenitors in multiple myeloma bone marrow. Clin Cancer Res. 2012;18(22):6155‐6168. 22988056 10.1158/1078-0432.CCR-12-0531 PMC3500436

[9] Kellner J , Wallace C , Liu B , Li Z . Definition of a multiple myeloma progenitor population in mice driven by enforced expression of XBP1s. JCI Insight. 2019;4(7).e124698. 10.1172/jci.insight.124698 PMC6483640 30944260

[10] Yan Z , Cao J , Cheng H , et al. A combination of humanised anti‐CD19 and anti‐BCMA CAR T cells in patients with relapsed or refractory multiple myeloma: a single‐arm, phase 2 trial. Lancet Haematol. 2019;6(10):e521‐e529. 31378662 10.1016/S2352-3026(19)30115-2

[11] Rajkumar SV , Dimopoulos MA , Palumbo A , et al. International Myeloma Working Group updated criteria for the diagnosis of multiple myeloma. Lancet Oncol. 2014;15(12):e538‐e548. 25439696 10.1016/S1470-2045(14)70442-5

[12] Lee DW , Gardner R , Porter DL , et al. Current concepts in the diagnosis and management of cytokine release syndrome. Blood. 2014;124(2):188‐195. 24876563 10.1182/blood-2014-05-552729 PMC4093680

[13] Chen AP , Setser A , Anadkat MJ , et al. Grading dermatologic adverse events of cancer treatments: the common terminology criteria for adverse events version 4.0. J Am Acad Dermatol. 2012;67(5):1025‐1039. 22502948 10.1016/j.jaad.2012.02.010

[14] Ghermezi M , Li M , Vardanyan S , et al. Serum B‐cell maturation antigen: a novel biomarker to predict outcomes for multiple myeloma patients. Haematologica. 2017;102(4):785‐795. 28034989 10.3324/haematol.2016.150896 PMC5395119

[15] Zhao W‐H , Liu J , Wang B‐Y , et al. A phase 1, open‐label study of LCAR‐B38M, a chimeric antigen receptor T cell therapy directed against B cell maturation antigen, in patients with relapsed or refractory multiple myeloma. J Hematol Oncol. 2018;11(1):141. 30572922 10.1186/s13045-018-0681-6 PMC6302465

[16] Sato S , Ono N , Steeber DA , Pisetsky DS , Tedder TF . CD19 regulates B lymphocyte signaling thresholds critical for the development of B‐1 lineage cells and autoimmunity. J Immunol. 1996;157(10):4371‐4378. 8906812

[17] Bradbury LE , Kansas GS , Levy S , Evans RL , Tedder TF . The CD19/CD21 signal transducing complex of human B lymphocytes includes the target of antiproliferative antibody‐1 and Leu‐13 molecules. J Immunol. 1992;149(9):2841‐2850. 1383329

[18] Loken MR , Shah VO , Dattilio KL , Civin CI . Flow cytometric analysis of human bone marrow. II. Normal B lymphocyte development. Blood. 1987;70(5):1316‐1324. 3117132

[19] Matsui W , Wang Q , Barber JP , et al. Clonogenic multiple myeloma progenitors, stem cell properties, and drug resistance. Cancer Res. 2008;68(1):190‐197. 18172311 10.1158/0008-5472.CAN-07-3096 PMC2603142

[20] Matsui W , Huff CA , Wang Q , et al. Characterization of clonogenic multiple myeloma cells. Blood. 2004;103(6):2332‐2336. 14630803 10.1182/blood-2003-09-3064 PMC3311914

[21] Hosen N , Matsuoka Y , Kishida S , et al. CD138‐negative clonogenic cells are plasma cells but not B cells in some multiple myeloma patients. Leukemia. 2012;26(9):2135‐2141. 22430638 10.1038/leu.2012.80

[22] Garfall AL , Stadtmauer EA , Hwang W‐T , et al. Anti‐CD19 CAR T cells with high‐dose melphalan and autologous stem cell transplantation for refractory multiple myeloma. JCI Insight. 2018;3(8).e120505. 10.1172/jci.insight.120505 PMC5931130 29669947

[23] Yang Y , Kohler ME , Chien CD , et al. TCR engagement negatively affects CD8 but not CD4 CAR T cell expansion and leukemic clearance. Sci Transl Med. 2017;9(417):eaag1209. 29167392 10.1126/scitranslmed.aag1209 PMC6944272

[24] Torikai H , Reik A , Liu P‐Q , et al. A foundation for universal T‐cell based immunotherapy: T cells engineered to express a CD19‐specific chimeric‐antigen‐receptor and eliminate expression of endogenous TCR. Blood. 2012;119(24):5697‐5705. 22535661 10.1182/blood-2012-01-405365 PMC3382929

[25] Zhang L , Tai Y‐T , Ho M , et al. Regulatory B cell‐myeloma cell interaction confers immunosuppression and promotes their survival in the bone marrow milieu. Blood Cancer J. 2017;7(3):e547. 28338671 10.1038/bcj.2017.24 PMC5380908

[26] Kessel A , Haj T , Peri R , et al. Human CD19(+)CD25(high) B regulatory cells suppress proliferation of CD4(+) T cells and enhance Foxp3 and CTLA‐4 expression in T‐regulatory cells. Autoimmun Rev. 2012;11(9):670‐677. 22155204 10.1016/j.autrev.2011.11.018

[27] Floudas A , Amu S , Fallon PG . New insights into IL‐10 dependent and IL‐10 independent mechanisms of regulatory B cell immune suppression. J Clin Immunol. 2016;36(Suppl 1):25‐33. 27008462 10.1007/s10875-016-0263-8

