# Supplementary material for: Sequential CD19 and BCMA‐specific CAR T‐cell treatment elicits sustained remission of relapsed and/or refractory myeloma
Source: Cancer Med. 2020 Dec 23;10(2):563–74. doi: 10.1002/cam4.3624 (PMC7877347; doi:10.1002/cam4.3624)
Supplement: Supplementary file 1 — Supplementary Material [file CAM4-10-563-s001.docx]

**Supplementary Method**

**Manufacturing GMP grade BCMA/CD19-CAR T-cell**

Autologous or allologous peripheral blood lymphocytes underwent density gradient separation from patients’ peripheral blood collected by apheresis. Subsequently, T cells were purified by anti-CD3 coated microbeads (Miltenyi Biotec, Bergisch Gladbach, Germany) and expanded with the complete medium [x-vivo 15 (Lonza, Alpharetta, GA, USA), 5% heat-inactivated autologous serum, 100 IU/mL IL-2, 2 mM GlutaMax (Life Technologies, Carlsbad, CA, USA) ] added 100 ng/mL anti-CD3 and 400 ng/mL anti-CD28 (Miltenyi Biotec, Bergisch Gladbach, Germany) for activation under a 37⁰C, 5% CO2 humidified condition. After activation for 48 hours, anti-BCMA/anti-CD19 vector supernatant was thawed and diluted with the complete media and incubated cell in Retronectin (Takara Bio Inc, Japan) -coated flask for 2 hours. After transduction, cells were concentrated and re-suspended in complete medium. The transduction process was duplicated at day 3. Transduced cells were maintained at a concentration from 0.5~1.2×10^6^ cells/mL in complete medium until day 12~20. Cells were harvested and concentrated in 0.9% saline solution (Sanlian, Haerbin, China) with 4% human serum albumin (Baxter, Westlake Village, CA, USA) for infusion. Infusion dose was 0.5~2 ×10^7^ CAR/CD3 double positive cells per kg of patient bodyweight.

Quality assessment of CAR T cell generation was conducted at the end of manufacture process. The amplified folds of total cell number ranged from 100 to 400, while the overwhelming majority of products (more than 96%) expressed CD3. The detection by flow cytometry indicated that the purity of anti-BCMA/anti-CD19 CAR expressed cell ranged from 30~70% in total cell population. Acceptance criteria for cell infusion were as follows:

1. Trypan Blue Viability > 90%;
2. Gram stain: Negative;
3. Sterility: Negative;
4. Mycoplasma (PCR): Negative;
5. CAR transduction efficiency > 10%;
6. Purity of CD3+ cell > 95%;
7. Endotoxin < 5 EU/mL;
8. CD19+ cell: Negative

It was determined that 50.12±14.45% cells expressed CAR-CD19 and 36.06±9.40% cells expressed CAR-BCMA in final products by flow cytometry (Suppl. Fig. 1A). CART-CD19 or CART-BCMA exhibited a specifically cytotoxic activity against CD19 or BCMA-expressing cells *in vitro* determined by LDH leakage assay (Suppl. Fig. 1B). The dramatically upgrade concentration of IFN-γ, IL-2 and TNF-α instead of IL-4, IL-6 and IL-10 in supernatants demonstrated the coculture elicited intensive Th1/Tc1 responses of CART-CD19 or CART-BCMA to target cells *in vitro.* (Suppl. Fig.1C).

**Suppl. Fig. 1**. Specific response of CART-CD19 or CART-BCMA to CD19 or BCMA expressed tumor cell in vitro. (A) CAR-CD19 or CAR-BCMA transgene expression on T cells of patient 01 was detected by immunostaining with PE-conjugated Protein-L. The sub-population is gated on CD3+ positive cells. (B) CART-CD19 or CART-BCMA cells were co-cultured with CD19 or BCMA-expressing target tumor cells (K562) in the absence of exogenous cytokines. The control was normal T cells with target cells. Specific cytotoxic activity of CART cells against target cells was detected using the lactate dehydrogenase release assay. (C) One day after co-culture with target cells, supernatant samples were collected and subjected to cytokine production assays using the Cytometric Bead Array (CBA) Kit.


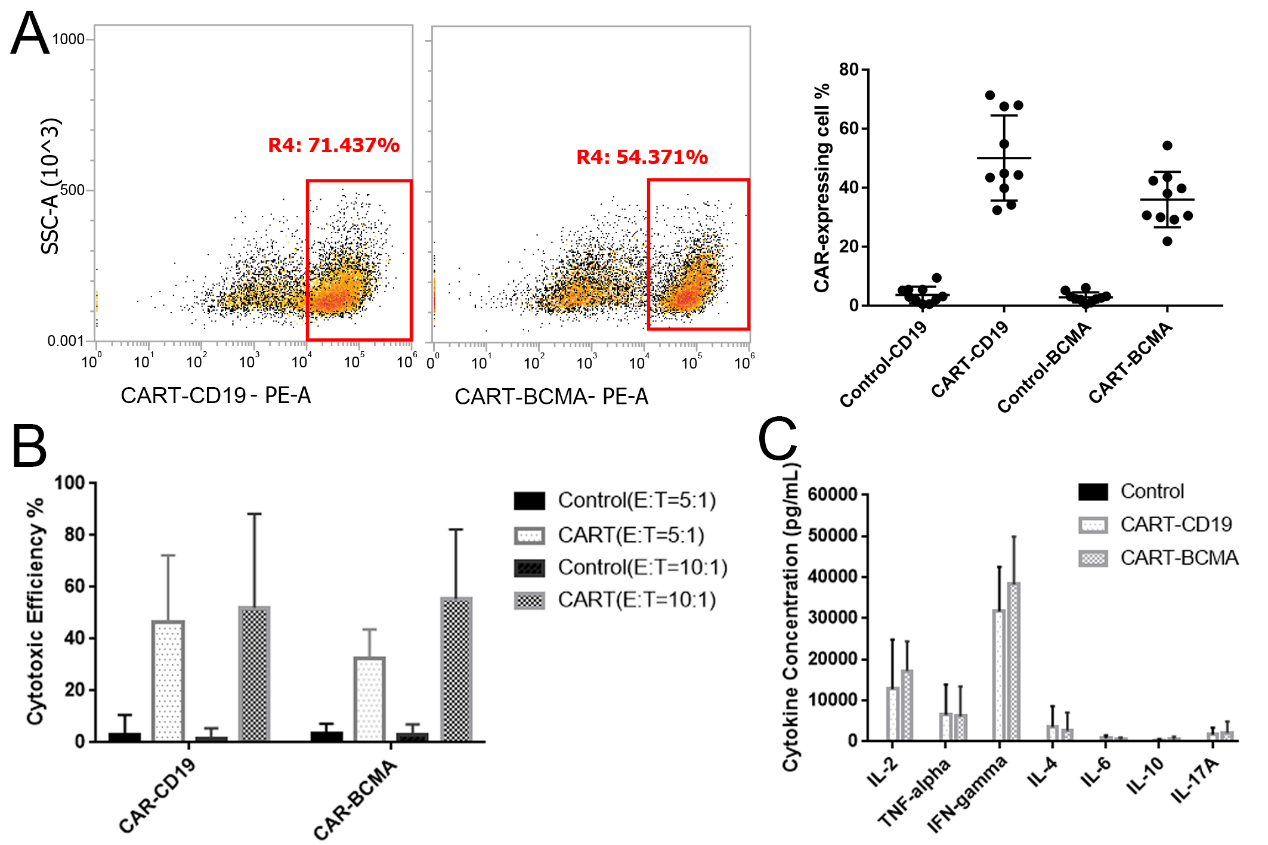


**Construction of chimeric antigen receptor lentiviral vector**

The anti-CD19 scFv-CD28-CD3ζ-IL-6 shRNA casstte lentiviral vector and anti-BCMA scFv-CD28-CD3ζ lentiviral vector were designed and manufactured by the Unicar-Therapy Bio-medicine Technology Company basing on previously reported vectors (Imai, C. 2004; Carpenter, R. O. 2013). Chimeric antigen receptor constructs in the study were present in a schematic diagram (Suppl. Fig 2.).

**Suppl. Fig. 2.** Construction of recombinant DNA sequence of chimeric antigen receptor.


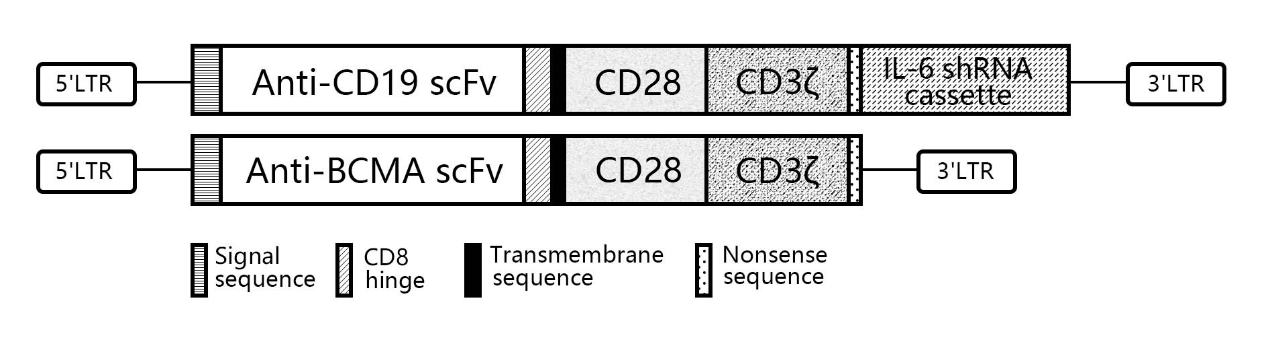


**DNA isolation and Q-PCR analysis**

Patients’ PBMC DNA was extracted by using a blood genomic DNA extraction kit (Tiangen, Beijing, China). PBMC DNA was amplified with a primer pair (Forward, CCTTTCCGGGACTTTCGCTTT; Reverse, GCAGAATCCAGGTGGCAACA) specific for CAR sequence. Real-time quantitative PCR was carried out with an ABI7500 PCR system (Applied Biosystems, Foster City, CA, USA). The approach to calculate CAR transgene copies in blood cell DNA was similar to that described previously (Grupp, S. A. 2013). A 5-point standard curve was generated consisting of 10^7^ to10^3^ copies lentivirus plasmid with 100 ng control genomic DNA to quantitative determine copy number. Copies transgene/per cell were calculated according to the formula: copies calculated from CD19 standard curve/detected copies/input DNA x 6.02 pg DNA/male somatic cell x CF.

**Suppl. Fig. 3.** Regulatory B cell repression in myeloma milieu is associated with clinical response against myeloma. The frequency of regulatory B cells (CD19+CD24+CD38+ population) within the peripheral blood and BM pre- and post-CART therapy for patient 05 was evaluated by flow cytometry.


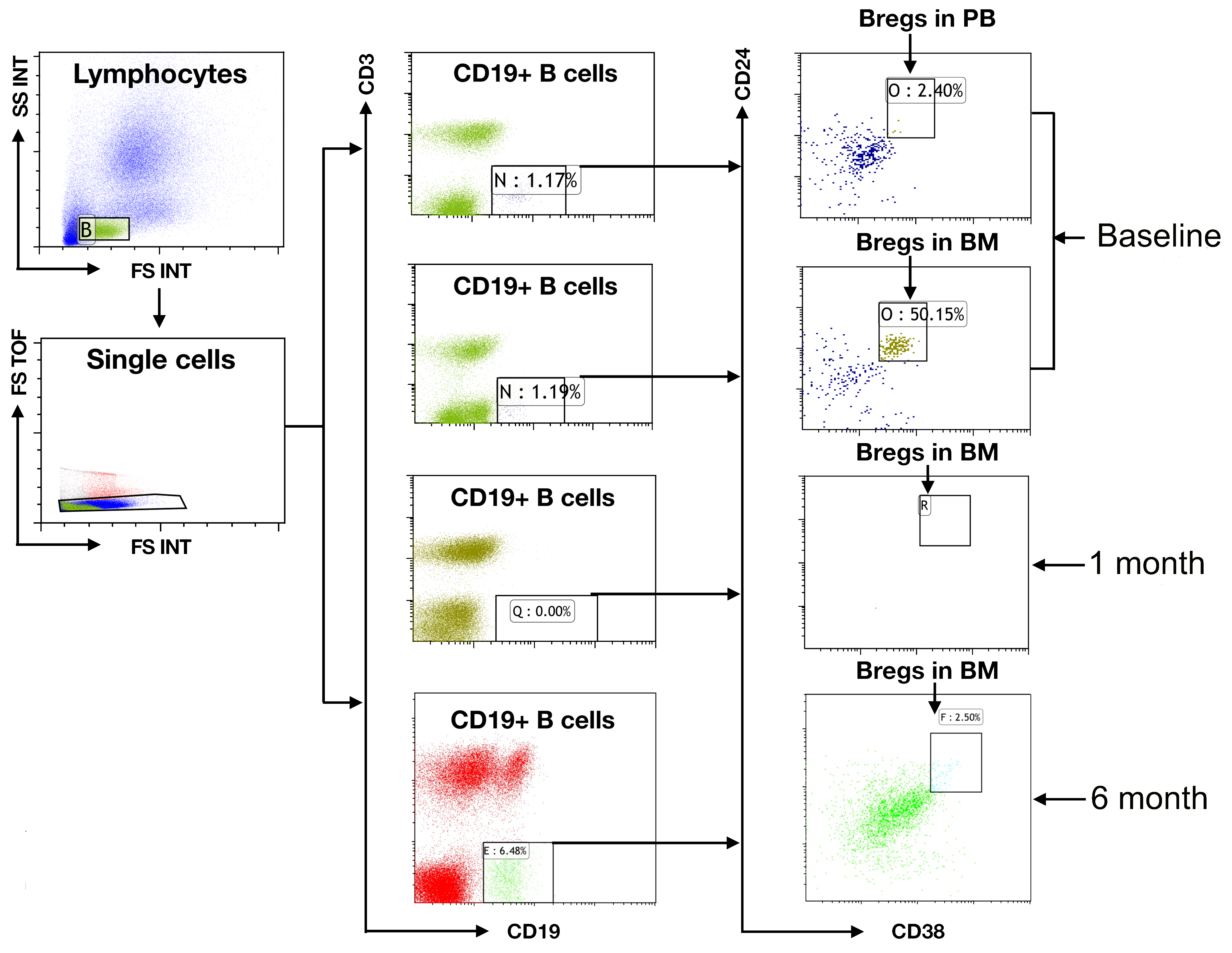


**Suppl. Tab. 1.** Previous new drug exposure and resistance prior to CART treatment

| Patient | Bortezomib | Carfilzomib | Ixazomib | Thalidomide | Lenalidomide | Pomalidomide |
| --- | --- | --- | --- | --- | --- | --- |
| 01 | +^*^ | NE^†^ | + | + | + | NE |
| 02 | + | NE | NE | + | + | NE |
| 03 | NE | NE | NE | + | NE | NE |
| 04 | + | NE | NE | + | NE | NE |
| 05 | + | NE | NE | + | NE | NE |
| 06 | + | NE | NE | + | + | NE |
| 07 | + | NE | NE | + | + | NE |
| 08 | + | + | + | + | + | + |
| 09 | + | NE | + | + | + | NE |
| 10 | + | NE | NE | + | NT^‡^ | NE |

*+: exposed and refractory (defined as “progression on or within 60 days of last dose of therapy or failure to achieve at least a minimal response after at least two full cycles of therapy”)

†NE: not exposed

‡NT: not tolerable due to severe bone marrow suppression

**Suppl. Tab. 2.** Acute and Late adverse events after CD19 and BCMA-CART cell infusion

| Toxicity | Acute (Within 2 weeks after infusion) | | | | Late (later than 2 weeks after infusion) | | | |
| --- | --- | --- | --- | --- | --- | --- | --- | --- |
|  | Hematological | | Non-hematological | | Hematological | | Non-hematological | |
| Grade | ≤2 | ≥3 | ≤2 | ≥3 | ≤2 | ≥3 | ≤2 | ≥3 |
| Patient 01*(First infusion) | Anemia Grade 1  Platelet↓Grade 1 | WBC ↓Grade 4  Neutrophil ↓ Grade 4  Lymphocyte ↓ Grade 4 | CRS Grade 2  Fever Grade 2  Hypotension Grade 2  Nausea Grade 2  NT-proBNP↑Grade 2  Serum creatine↑Grade 2  Hypoxia Grade 2  Diarrhea Grade 1  Redness flush Grade 1  Weakness and muscular soreness Grade 1  APTT prolonged Grade 1  Fibrinogen↓Grade 1  Blood bilirubin↑Grade 1  GGT↑Grade 1  Hypokalemia Grade 1 | TLS Grade 3 | None | None | Blood bilirubin↑Grade 2  ALT↑Grade 2  AST↑Grade 2  Upper respiratory tract infection Grade 1 | None |
| Patient 01† (Second infusion) | WBC ↓Grade 2  Neutrophil ↓ Grade 2  Platelet↓ Grade 2  Anemia Grade 1 | Lymphocyte ↓ Grade 4 | CRS Grade 1  Fever Grade 1  Anorexia Grade 1  Weakness and muscular soreness Grade 1  Blood bilirubin↑Grade 1  ALT↑Grade 1  GGT↑Grade 1 | None | None | None | Blood bilirubin↑Grade 1  ALT↑Grade 1  Upper respiratory tract infection Grade 1 | None |
| Patient 02 | WBC↓ Grade 2  Neutrophil↓ Grade 2  Anemia Grade 1 | Lymphocyte↓ Grade 4  Platelet↓ Grade 3 | CRS Grade 1  Fever Grade 2  Weakness and muscular soreness Grade 1  NT-proBNP↑Grade 1  Hypokalemia Grade 1 | None | WBC ↓ Grade 2  Neutrophil ↓ Grade 2  Lymphocyte ↓ Grade 2  Platelet ↓ Grade 2 | None | Diarrhea Grade 1  Blood bilirubin↑Grade 1  Hypokalemia Grade 1  Hypogammaglobulinemia need IVIG support | None |
| Patient 03 | WBC↓Grade 2  Neutrophil↓Grade 2  Platelet↓Grade 2  Anemia Grade 1 | Lymphocyte↓Grade 4 | CRS Grade 2  Fever Grade 1  Hypoxia Grade 1  Anorexia Grade 1  Diarrhea Grade 1  Redness flush Grade 1  Weakness and muscular soreness Grade 1  serum creatine↑Grade 1  ALT↑Grade 1  GGT↑Grade 1 | None | WBC↓Grade 1  Lymphocyte↓Grade 1 | None | Upper respiratory tract infection Grade 1 | None |
| Patient 04 | WBC↓Grade 2  Neutrophil↓Grade 2  Anemia Grade 2  Platelet↓Grade 2 | Lymphocyte↓Grade 4 | CRS Grade 1  Fever Grade 1  Weakness Grade 1  NT-proBNP↑Grade 1 | None | Lymphocyte↓Grade 2  Anemia Grade 2 | WBC↓Grade 3  Neutrophil↓Grade 3  Platelet↓Grade 3 | Skin ulcer infection Grade 2  Blood bilirubin increased Grade 1 | None |
| Patient 05 | Neutrophil↓Grade 2  Anemia Grade 2 | Lymphocyte↓Grade 4  WBC↓Grade 3 | CRS Grade 1  Fever Grade 1  Redness flush Grade 1  Weakness Grade 1  NT-proBNP↑Grade 1 | None | Anemia Grade 1 | None | Respiratory infection Grade 2  Fever：Grade 1  Hypogammaglobulinemia need IVIG support | None |
| Patient 06* | Anemia Grade 2 | WBC↓Grade 4  Neutrophil↓Grade 4  Lymphocyte↓Grade 4  Platelet↓Grade 4 | Hypoxia Grade 2  Hypotension Grade 2  Nausea Grade 2  Vomit Grade 2  Diarrhea Grade 2  soreness Grade 2  Serum creatine↑Grade 2  Blood bilirubin↑Grade 2  Redness flush Grade 1  Headache Grade 1  Weakness and muscular  APTT prolonged Grade 1  GGT↑Grade 1  Hypokalemia Grade 1 | CRS Grade 3  Fever Grade 3  NT-proBNP↑Grade 3 | WBC↓Grade 2  Neutrophil↓Grade 2  Lymphocyte↓Grade 2  Anemia Grade 2  Platelet↓Grade 1 | None | Hypogammaglobulinemia: need IVIG support | None |
| Patient 07 | None | WBC↓Grade 4  Neutrophil↓Grade 4  Lymphocyte↓Grade 4  Anemia Grade 3  Platelet↓Grade 3 | CRS Grade 2  Nausea Grade 2  Weakness and muscular soreness Grade 2  APTT prolonged：Grade 2  Hypoxia Grade 1  Anorexia Grade 1  Vomit Grade 1  Diarrhea Grade 1  ALT↑Grade 1  ALP↑Grade 1  GGT↑Grade 1  Hypokalemia Grade 1 | Fever Grade 3  NT-proBNP↑Grade 3 | WBC↓Grade 1  Neutrophil↓Grade 1  Anemia Grade 1 | None | Respiratory infection Grade 2 | None |
| Patient 08 | None | Lymphocyte↓Grade 4  Platelet↓Grade 4  WBC↓Grade 3  Neutrophil↓Grade 3  Anemia Grade 3 | CRS Grade 2  CRES Grade 1  Dizziness Grade 2  Hypotension Grade 2  Nausea Grade 2  Vomit Grade 2  Weakness and Muscular soreness Grade 2  Sinus tachycardia Grade 2  Hypoxia Grade 1  APTT prolonged Grade 1  INR↑Grade 1  Serum myoglobin Grade 1  Blood bilirubin↑Grade 1  Hypokalemia Grade 1 | Fever Grade 4  NT-proBNP↑Grade 3 | WBC↓Grade 2  Neutrophil↓Grade 2  Lymphocyte↓Grade 2  Anemia Grade 2  Platelet↓Grade 2 | None | Diarrhea Grade 1  Blood bilirubin↑Grade 1  Hypokalemia：Grade 1  Hypogammaglobulinemia: need IVIG support | None |
| Patient 09 | None | Lymphocyte↓Grade 4  WBC↓ Grade 3  Neutrophil↓Grade 3  Anemia Grade 3  Platelet↓Grade 3 | CRS Grade 2  Fever Grade 2  Hypoxia Grade 2  Hypotension Grade 2  Nausea Grade 2  Sinus tachycardia:Grade 2  Serum creatine↑Grade 2  soreness Grade 2  Hypokalemia Grade 2  Redness flush Grade 1  Catheter derived staphylococcal septicemia Grade 1  Conjunctival bleeding Grade 1  Dyspnea Grade 1  Vomit Grade 1  Diarrhea Grade 1  Weakness and muscular  GGT↑Grade 1  Hypoalbuminemia Grade 1  Hyponatremia Grade 1  Lipase↑Grade 1 | TLS Grade 3  NT-proBNP↑Grade 3 | WBC↓Grade 1  Neutrophil↓Grade 1  Platelet↓Grade 1 | Anemia Grade 3 | None | None |
| Patient 10 | Anemia Grade 2  Platelet↓Grade 2 | Neutrophil↓Grade 4  Lymphocyte↓Grade 4  WBC↓Grade 3 | CRS Grade 1  Fever Grade 2  Weakness Grade 1  NT-proBNP↑Grade 1  ALT↑Grade 1  ALP↑Grade 1  GGT ↑ Grade 1  Hypokalemia Grade 1 | None | WBC↓Grade 2  Neutrophil↓Grade 2  Lymphocyte↓Grade 2  Anemia Grade 1  Platelet↓Grade 1 | None | ALT↑Grade 1 | None |

Note: CRS was graded according to recommendations by Lee et al and CRES was graded according to recommendations of the CAR-T cell-therapy-associated toxicity (CARTOX) Working Group. Other toxicities were assessed according to the National Cancer Institute Common Terminology Criteria for Adverse Events, version 4.03. Adverse events were ranked from highest to lowest by grade. All grade adverse events within the first 2 weeks and more than 2 weeks after CAR T-cell infusion are listed. ↑indicates cell count or lab test value increased, ↓indicates cell count or lab test value decreased. * Patient 01 and patient 06 received one dose immunosuppression interventions of tocilizumab 4mg/kg. All other patients did not receive immunosuppressive intervention including tocilizumab and glucocorticoid. † Patient 01’s adverse events were recorded post first infusion with CART-BCMA and post second infusion with CART-CD19 and CART-BCMA respectively.

Abbreviations: AE, adverse event; WBC, white blood cell; ALT, alanine aminotransferase ; AST, aspartate aminotransferase ; GGT, γ-glutamyl transpeptadase ;ALP: alkaline phosphatase; NT-proBNP, N-Terminal pro-brain natriuretic peptide ; APTT, activated partial thromboplastin time; CRS, cytokine release syndrome; CRES, CAR‑T‑cell‑related encephalopathy syndrome
